# Relaxation time mapping technique development improves disease detectability

**DOI:** 10.1186/1532-429X-18-S1-W37

**Published:** 2016-01-27

**Authors:** Walter R Witschey, Jing Wang, Harold Litt, Yuchi Han

**Affiliations:** 1 Medicine, University of Pennsylvania, Philadelphia, PA USA; 2 Radiology, University of Pennsylvania, Philadelphia, PA USA

## Background

Relaxation time mapping is increasingly important for myocardial tissue characterization and potentially offers a quantitative descriptor of occult or heterogeneous myocardial disease. Recent techniques that reduce motion sensitivity, image artifacts and correct for elevated heart rates (HR) have made substantial advances in map accuracy and precision, but it is unclear how these improvements quantitatively lead to better disease detection. We used T1rho maps from normal subjects to generate variance-matched Monte Carlo data and retrospectively analyzed the effect that incremental improvements in mapping technology have on disease detectability.

## Methods

T1rho MRI data was acquired in 14 normal subjects using a 2D single-shot T1rho-prepared balanced steady-state free-precession sequence with a 90_x_-SL_y_-180_y_-SL_-y_-90_-x_ pulse cluster with 8 images (TSL = 2-50 ms and B1 = 500 Hz) at 1.5 T. Motion correction (MoCo) was performed using an optical flow algorithm. HR correction was performed with an initial dummy scan to presaturate the longitudinal magnetization. Uncorrected, HR corrected, MoCo, and dual MoCo and HR corrected images were contoured and mean relaxation times were estimated using a 6 segment model (QMass, Medis). Monte Carlo simulations (10k trials) were performed with matched-variance and true disease scores at ΔT1rho = +5,10 and 15 ms. For per-patient disease detectability, ROC curves were generated for each level. For study-based group disease detectability, 1-way ANOVA was performed with Bonferroni correction.

## Results

**Figure 1:**
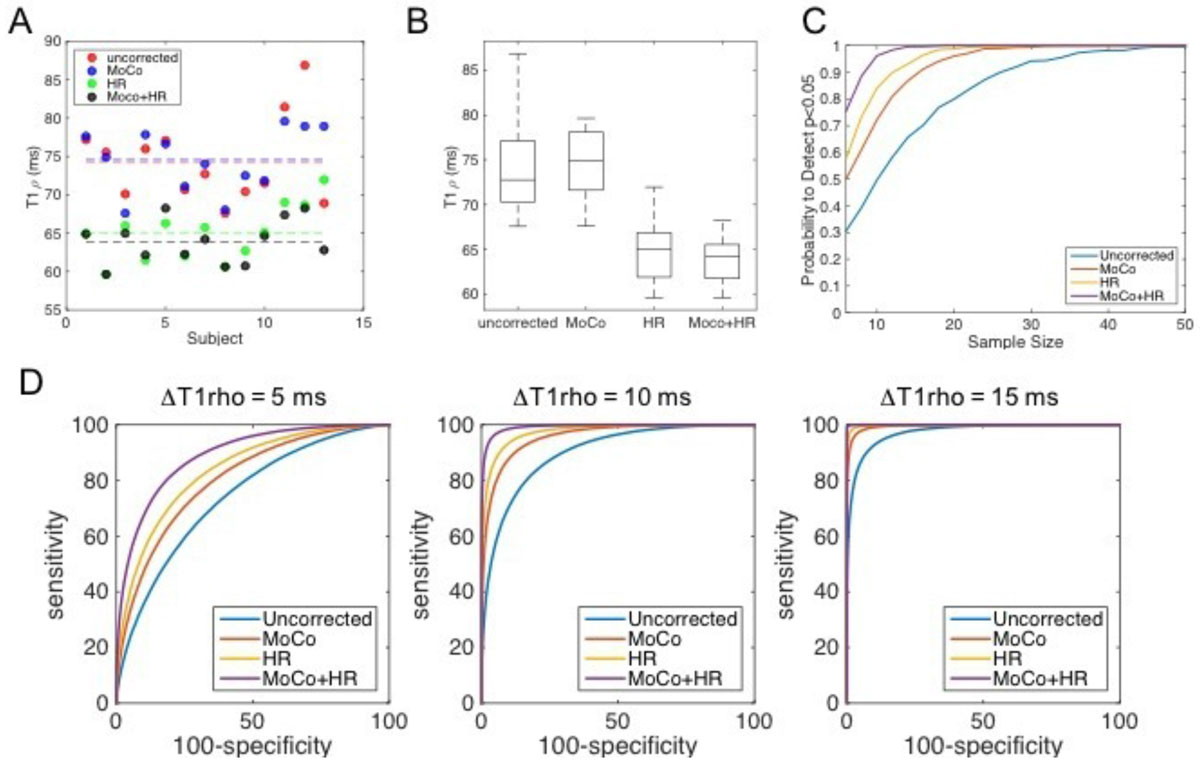
Figure 1

MoCo mainly reduced relaxation time variance from T1rho = 74.3 ± 5.5 to 74.6 ± 4.1 ms and HR correction minimized both bias and variance to 64.9 ± 3.6 ms. Together, MoCo and HR correction were 63.9 ± 2.9 ms (Figure 1A and 1B). Figure 1C shows the probability to detect ΔT1rho = 5 ms (p < 0.05) for different sample sizes. MoCo and HR incrementally increase this probability substantially and joint correction achieves high levels of disease detectability at N = 10 subjects per group (>90% detection rate). MoCo and HR correction had independent, incremental and substantial improvements in disease detectability as shown by the progressive improvement in ROC (Figure 1D). While these findings are applicable to other types of mapping techniques, a limitation is that equal variance was assumed among groups.

## Conclusions

In conclusion, the Monte Carlo methods introduced here demonstrate that incremental improvements in mapping techniques by MoCo and HR correction have substantial improvements in disease detectability. This will be important for future technique development performance comparisons, myocardial disease detection in individual patients and for correct sample size determination in clinical trials.

